# Transcriptome-based identification and validation of reference genes for corm growth stages, different tissues, and drought stress in Taro (*Colocasia esculenta*)

**DOI:** 10.1186/s12870-024-05199-x

**Published:** 2024-05-30

**Authors:** Weiqing Dong, Qi Chen, Fanglian He

**Affiliations:** 1 Vegetable Research Institute, Guangxi Zhuang Autonomous Region Academy of Agricultural Sciences, Nanning, 530007 China; 2 New Technology Entrepreneur Center, Nanning, 530007 China

**Keywords:** Reference gene, Taro, Corm growth, RT-qPCR

## Abstract

Taro is a widely utilized starch resource plant. It is essential to quantify the expression levels of functional genes associated with taro growth using real-time quantitative polymerase chain reaction (RT-qPCR). However, to obtain reliable RT-qPCR results, appropriate reference genes (RGs) are required for data normalization. In this study, we screened seven novel candidate RGs using transcriptome datasets from taro, encompassing data from growth corms and various tissues. The expression stability of these seven new RGs, along with the commonly used RGs *Actin*, *EF1-α*, and *β-tubulin*, was assessed using Delta Ct, BestKeeper, geNorm, and NormFinder algorithms. Furthermore, we conducted a comprehensive analysis using the RefFinder program and validated the results using the target gene, *CeAGPL1*. The findings revealed that *ACY-1* and *PIA2* were the optimal multiple RGs for normalization during corm growth, while *COX10* and *Armc8* were suitable for samples including various types of tissues. Furthermore, we found three RGs, Armc8, *COX10* and *CCX4L*, were the optimal RGs for drought stress. This study assessed the suitability of RGs in taro for the first time. The identified RGs provide valuable resources for studying corm growth, diverse tissues, and drought stress. This study contributes to the advancement of our understanding of the underlying mechanisms that govern the growth of taro.

**Supplementary Information:**

The online version contains supplementary material available at 10.1186/s12870-024-05199-x.

## Introduction

Taro (*Colocasia esculenta*) is a starchy root crop that has been cultivated and consumed by humans for thousands of years [1]. It is a tropical and subtropical plant that belongs to the Araceae family and is widely grown in many countries around the world, including Asia, Africa, the Caribbean, and the Pacific Islands [2, 3]. Taro is known by various names in different regions, such as "dasheen" in the Caribbean [3], "kalo" in Hawaii [4], "arvi" in India [5], and "eddo" in Japan [6]. Taro is a staple food for many indigenous communities and has played a significant role in their cultural and dietary practices. It is a versatile crop that can be used in a variety of culinary applications, such as boiling, frying, baking, and mashing. Taro leaves are edible and are commonly used in traditional dishes. In addition to its culinary importance, taro has been used in traditional medicine because of its potential health benefits [7]. Apart from its cultural and dietary significance, taro is also economically important as a cash crop. It is a source of income for many small-scale farmers in developing countries, and the global trade in taro and taro-derived products has been steadily increasing. Taro is gaining popularity in modern cuisines because of its unique flavor, nutritional value, and potential as a gluten-free alternative to wheat-based products [8].

Taro corm, which is the edible part, contains high levels of carbohydrates, fats, crude fiber, vitamin C, thiamin, riboflavin, and niacin [9]. With a rich carbohydrate content and energy value of 135 kcal/100 g, taro corms have almost double the carbohydrate content of potatoes, and their protein content is 11% higher than that of yams, cassava, and sweet potatoes [10]. The growth of taro corm occurs in three phases. Given the nutrient-rich profile of taro corms, it is important to understand their genetic basis for the corm growth, particularly the regulation of pathways related to carbohydrate accumulation [11, 12].

Despite its importance, research on taro has been limited compared to that on other major crops. However, with advancements in molecular biology and genomics, there has been increasing interest in studying taro at the molecular level to understand the genetics, physiology, and molecular pathways associated with important traits, such as yield, disease resistance, and nutritional content. One crucial aspect of molecular research is the validation of reference genes (RGs), which are used as internal controls for normalizing gene expression data in quantitative gene expression studies, such as reverse transcription quantitative polymerase chain reaction (RT-qPCR). *Actin* was used as a control gene to determine the transcript levels of a metal-responsive gene, *pCeMT*, in *C. esculenta* [13]. Lekshmi et al. used *actin* as a RG to quantify the expression of resistant gene analogue in taro with qPCR [14]. In additional, Wang et al. normalized the expression of multiple genes during cold storage using the *actin7* gene [15]. In a transcriptome study of taro corm growth, *actin* was selected as an RG to verify the transcriptome. Obviously, researchers tended to use *actin* as RG in taro studies. Considering there is no study on the stability of RG in taro, we supposed that this may be due to *actin* being a widely used RG. However, it has become evident in some experimental reports that conventional RG, such as *actin*, is unsuitable owing to its inherent variability [16–18]. To date, no studies have been published on the validation of RGs for RT-qPCR in taro.

**Fig. 1:**
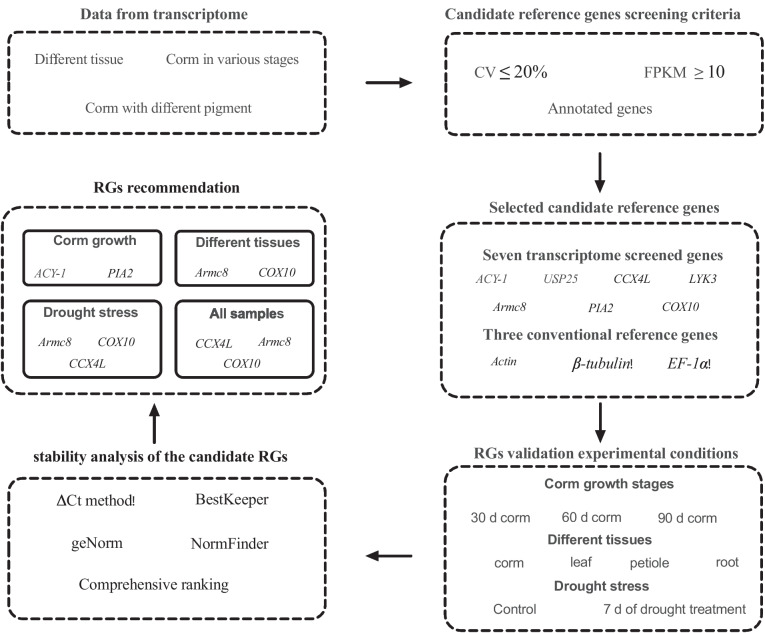
The comprehensive strategy of this study

Proper validation of RGs is crucial to ensure the accuracy and reliability of gene expression data, as their expression levels should remain stable under different experimental conditions and across different tissues and growth stages. Therefore, the validation of RGs in taro is a critical step towards understanding its gene expression regulation, identifying key genes associated with important traits, and improving breeding strategies for taro improvement. In this study, we aimed to validate a set of RGs for taro and to provide a reliable toolkit for gene expression studies in this important crop. The findings of this study will contribute to the advancement of taro research and provide valuable insights for the scientific community and stakeholders involved in taro production and utilization (Fig. 1).

## Materials and methods

### Candidate RG screening

To identify suitable RGs for corm growth and different tissues in taro, we employed gene expression value obtained derived from a total of 42 transcriptome datasets from various studies. These datasets encompassed information related to corm color (PRJNA639211) [19], corm growth (PRJNA756190) [20], and four kinds of tissues (Unpublished). The FPKM value (Fragments per kilobase per million mapped reads) of each gene was obtained using StringTie software for gene stable analysis [21, 22]. To assess the expression stability of each gene, we computed several indices based on the FPKM values using Excel software. These indices included the mean FPKM value (MF), standard deviation (SD), and coefficient of variation (CV) value, which is obtained by dividing the SD by the MF, according to the methods described previously [23]. The genes with credible annotation and relative high expression (MF ≥ 10) were selected based on the CV value (≤ 20%).

### Plant materials

The taro variety "Lipuyu No. 2" (Colocasia esculenta L. Schott) were planted in field at Guangxi Academy of Agricultural Sciences, Nanning, Guangxi, China. Taro corms at different stages of growth, namely one, two, and three months old, were harvested separately. The leaf, corm, petiole, and root samples were collected at the stage of three months. For drought stress, the samples of three months old were exposed to a seven-days water deficit treatment according to the previous study [24]. After harvesting, the samples were thoroughly washed with running water and then with distilled water. Subsequently, the samples were rapidly frozen in liquid nitrogen and stored in a − 80 °C refrigerator until they were processed for RNA extraction. At each sampling time, three samples were collected from three different plants to ensure proper representation for subsequent analysis.

### RNA extraction, cDNA synthesis, and qPCR

Total RNA was extracted using TRIzol Reagent (Invitrogen, Carlsbad, CA, United States) following the manufacturer's instructions. To assess the quality and integrity of the extracted RNAs, agarose electrophoresis and NanoDrop 2000 spectrophotometer (Thermo Scientific, United States) were employed, respectively. To mitigate the risk of DNA contamination, the resulting RNAs underwent treatment with DNase to degrade any residual genomic DNA. Subsequently, one microgram of RNA was utilized for first-strand cDNA synthesis using the HiScript III RT SuperMix for qPCR (Vazyme, China). The ChamQ SYBR qPCR Master Mix (Vazyme, China) was employed for the qPCR reaction. The qRT-PCR assays were conducted in a 25-μL reaction mixture comprising 12.5 μL of Master Mix, 1.0 μL each of forward and reverse primers (0.4 μM), 2 μL of template cDNA (< 100 ng), and 8.5 μL of nuclease-free water. The quantitative amplification was performed using a qTOWER Real-time Thermal Cycler (analytikjena, Germany) with the following cycling conditions: an initial cycle at 95 °C for 30 s, followed by 40 cycles at 95 °C for 5 s, and 60 °C for 30 s. After each amplification, a melting curve analysis was conducted between 55 °C and 95 °C to confirm product specificity. Both no-template and no-RT controls were included for each primer pair. Each RT-qPCR experiment was performed in triplicate.

### Analysis of the expression stability of RGs

The expression levels of candidate RGs were assessed based on the cycle of threshold (Ct) value. To determine the expression stability of these candidate RGs, four statistical algorithms were employed: Delta Ct method [25], BestKeeper [26], geNorm [27], and NormFinder [28]. For BestKeeper analysis, the raw Ct values were directly utilized, while for the other two analyses, the raw data were transformed into relative quantities using the 2^^−ΔCt^ method (where ΔCt = eachCt—minimumCt). To ensure the proper functioning of the four algorithms and to obtain a comprehensive ranking of RGs from the experimental data, the online tool RefFinder was employed [29]. This tool integrated the outputs from the three algorithms, providing a reliable and consolidated assessment of the expression stability of the candidate RGs.

### Validation of optimal RGs

To validate the selected RGs, the expression level of a target gene, *CeAGPL1* (GeneBank: OK544528), was performed using the same RNA samples employed for RG selection. The *CeAGPL1* encode the large subunit of ADP-glucose pyrophosphorylase, which is upregulated in the corm and increases its expression as corm growth progresses [30]. cDNA synthesis and qPCR were conducted under the same conditions as described above. The relative expression level of the *CeAGPL1* gene was calculated using the 2^^−ΔΔCt^ method [31]. For data normalization, three RG strategies were applied, including: The optimal multiple RGs from each treatment, the most stable RG from each treatment, and the least stable RG. To assess statistical differences, the expression value was subjected to two-way ANOVA using Sidak's test at a significance level of p < 0.05 with a Prism software. For robustness, three replicates of four independent experiments were performed, ensuring reliable and consistent results in the validation process.

## Results

### Selection of candidate RGs based on the transcriptome data

To select RGs with minimal expression variation for RT-qPCR experiments, we performed a prior analysis of gene stability utilizing RNA-seq data obtained from taro tissues at various stages and treatments. The FPKM values of all transcripts in each taro sample were acquired from transcriptome datasets. The coefficient of variation (CV) value of FPKM for each gene across all samples was calculated and subsequently sorted in ascending order. The gene exhibiting the lowest CV value was chosen as the most promising RG. A total of 14 genes had a CV value lower than 20%. Furthermore, genes should have an average FPKM value higher than 10. Among them, seven annotated genes were selected for analysis of expression stability (Table 1 and Supplementary Table S1). Additionally, three commonly used RGs, namely *actin*, *β-tubulin*, and *EF-1α*, were included.

**Table 1:** The summary information of candidate RGs in taro based on the transcriptome data

Genes symbol	Gene ID	Mean FPKM	SD	CV (%)
*ACY-1*	Taro_009007	34.95	6.22	17.80
*USP25*	Taro_020373	12.05	2.17	17.98
*CCX4L*	Taro_006024	18.09	3.46	19.13
*LYK3*	Taro_011247	15.34	2.96	19.28
*PIA2*	Taro_008077	65.07	12.85	19.75
*Armc8*	Taro_049522	12.15	2.40	19.78
*COX10*	Taro_016205	22.46	4.52	20.14

### Specificity and expression analysis of the candidate RGs

**Fig. 2:**
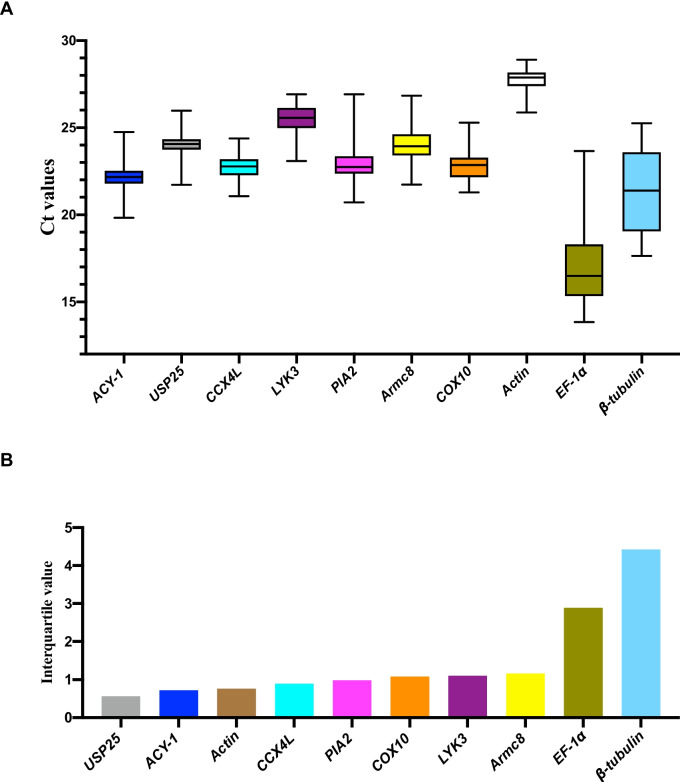
RT-qPCR Ct values and interquartile ranges are shown. **A** Ct values of each RG in all samples are presented. The median is represented by a line across the box, while the box indicates the 25th and 75th percentiles. Whiskers represent the maximum and minimum values. **B** The interquartile ranges demonstrate the variability of Ct values between the 25th and 75th percentiles

To evaluate the specificity and expression profile of the 10 candidate RGs, primers were designed and used to amplify cDNA templates. The melting curves and threshold cycle (Ct) values were determined using RT-qPCR, which represent the specificity and expression level of each gene, respectively. Successful amplification yielded a single band of the expected size, indicating accurate target amplification. Melting curve analysis of primers of all seven candidate genes displayed a single peak, indicating strong primer specificity and the absence of non-specific amplification. The amplification product lengths ranged from 128 to 194 bp. To determine the amplification efficiency of the primers, a serial dilution of cDNA was used as qPCR templates, and the efficiency was calculated based on the slope of the standard curve using Ct values and template concentrations. The calculated efficiencies of the primers ranged from 94.5% to 105.2%. (Table 2, Supplementary Fig. 1). The expression levels of candidate RGs were determined using threshold cycle (Ct) values obtained from RT-qPCR (Supplementary table S2). A smaller Ct value indicated higher gene expression abundance, while a larger Ct value indicated lower abundance. The average Ct values of each gene ranged from 17.15 (*EF-1α*) to 27.78 (*Actin*), which meet the recommended values of higher than 15 and lower than 30 [32]. Based on the distribution of raw Ct values, *USP25*, *ACY-1*, *Actin*, *CCX4L*, and *PIA2* exhibited lower variability (Fig. 2A). However, commonly used RGs *EF-1α* and *β-tubulin* were considered unfavorable candidates due to their Ct values spanning multiple units, as observed in the interquartile range (Fig. 2B). Nevertheless, relying solely on the distribution of raw Ct values and the interquartile range is insufficient for assessing the expression stability of candidate RGs. Additional methods are necessary for more comprehensive and accurate evaluations.

**Table 2:** Primer sequences of 10 genes used for RG validation

Gene symbol	Gene description	Primer sequences	Product size (bp)	Amplification Efficiency	Notes
*ACY-1*	aminoacylase-1	AACAATGGCGAGGAGCAACA	150	99.1%	This study
		TATTGACGGAACCTGGAGATGG			
*USP25*	ubiquitin carboxyl-terminal hydrolase 25	CACGAGTTCCTCCGCTATGTT	178	105.2%	This study
		GGCACTTCACCTGGCTAAGAA			
*CCX4L*	cation/calcium exchanger 4-like	TTCCTAAGGTCGCATCCACAAT	164	96.5%	This study
		CAGAAGTAGTCCGCAGCAGTAT			
*LYK3*	lysM domain receptor-like kinase 3	GGCGATGGCAATCTGGTAGT	192	102.2%	This study
		GAGAGCGGAGAAGAGGATAGTG			
*PIA2*	phytochrome-interacting ankyrin-repeat protein 2	TGGCGGAAGTTGCTCGTT	128	95.8%	This study
		CAATGCTCTGGCGGATACATAG			
*Armc8*	armadillo repeat-containing protein 8	GACGAGAAGGTTGTGGATGCT	194	104.2	This study
		TTCTGCTCTGTGCTGGATTCA			
*COX10*	protoheme IX farnesyltransferase	TCTCCAGTCACCTCCATTCATC	157	96.8%	This study
		TCAGTAGCCTCTAACACCTTCC			
*Actin*	Actin	CCTTCGTCTTGATCTGGCAG	181	98.1%	[20]
		AGATGAGTTGGTCTTCGCAGTC			
*β-tubulin*	Beta-tubulin	GTAACCAGATCGGAGCCAAGT	156	99.5%	This study
		GCAGGAAGCCTCGTTGTAGTA			
*EF-1α*	Elongation factor 1-alpha	TGAGCGTGAGCGTGGTATC	131	94.5%	This study
		TCAATGATGAGGACAGCACAGT

### Expression stability analysis of the candidate RGs

The expression stability of 10 candidate RGs in the leaf, petiole, corm, and root tissues of taro was analyzed under various stages, including different corm growth stages, tissue types, drought stress, and across all samples. The analysis was performed using Delta Ct, BestKeeper, NormFinder, and geNorm methods (Supplementary table S3).

### Delta Ct method analysis

Using the Delta Ct method [25], the stability of genes was assessed by calculating the mean standard deviation (SD) of Delta Ct values between one gene and the other 9 genes. A lower mean SD indicates higher gene stability. Table 3 presents the ranking of RGs and their corresponding expression stability values during various growth stages and treatments. *PIA2* demonstrated the highest stability among RGs across different corm growth stages. Specifically, *COX10* exhibited the highest stability in various tissues and under drought stress treatment. In the overall sample set, *CCX4L* was identified as the most stable RG. Conversely, *β-tubulin* was consistently observed as the least stable RG, in different corm growth stages, tissues, or drought stress treatments.

**Table 3:** The RGs were ranked based on their suitability for normalization, and their expression stability values were determined using the Delta Ct method

**Stability Ranking**	**Corm growth**		**Different tissues**		**Drought stress**		**All samples**	
	**Gene**	**Aver. SD**	**Gene**	**Aver. SD**	**Gene**	**Aver. SD**	**Gene**	**Aver. SD**
1	*PIA2*	0.45	*COX10*	0.87	*COX10*	0.99	*CCX4L*	0.99
2	*ACY-1*	0.47	*PIA2*	0.87	*Armc8*	1	*Armc8*	1
3	*CCX4L*	0.48	*Armc8*	0.91	*CCX4L*	1	*COX10*	1
4	*USP25*	0.56	*USP25*	0.91	*PIA2*	1.06	*PIA2*	1.02
5	*COX10*	0.58	*CCX4L*	0.93	*USP25*	1.1	*ACY-1*	1.06
6	*LYK3*	0.58	*ACY-1*	0.99	*ACY-1*	1.13	*USP25*	1.09
7	*Armc8*	0.59	*Actin*	1.06	*LYK3*	1.31	*Actin*	1.27
8	*EF-1α*	0.63	*LYK3*	1.12	*Actin*	1.39	*LYK3*	1.36
9	*Actin*	0.7	*EF1_a*	1.96	*EF1_a*	1.9	*EF1_a*	1.85
10	*β-tubulin*	0.79	*β-tubulin*	2.13	*β-tubulin*	1.96	*β-tubulin*	2.3

### BestKeeper analysis

**Fig. 3:**
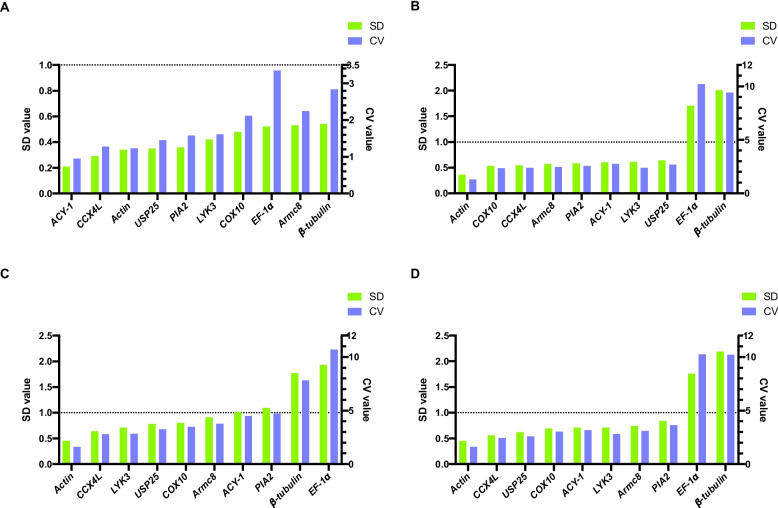
The expression stability of candidate reference genes was assessed using the BestKeeper program. The SD and CV value of each gene were shown in histogram, which represent the expression stability of genes during corm growth stages (**A**), in various tissues (**B**), under drought stress (**C**), and all the samples (**D**), respectively. SD stands for standard deviation, and CV represents the coefficient of variation. The lower value indicates more stable

The BestKeeper method, an Excel-based tool, utilizes pairwise correlations to assess the expression levels of candidate genes [26]. We calculated the standard deviation (SD) and coefficient of variance (CV) to evaluate the stability of 10 candidate RGs. The candidate RGs are ranked based on the two indexes with the most stable expression demonstrating the lowest SD value (Fig. 3). The analysis revealed that for corm growth stages, the optimal RG was *ACY-1*. *Actin* was identified as the most suitable RG for different tissues, drought stress treatment, and the total sample set. In terms of stability, the least reliable RG was *β-tubulin*, except for drought stress treatment, in which the most unstable gene was *EF-1α*.

### geNorm analysis

**Fig. 4:**
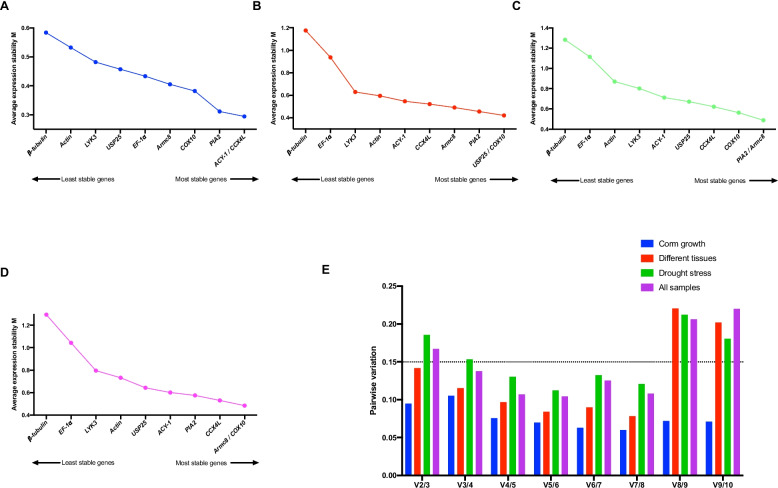
The stability and optimal number of RGs evaluated by geNorm software. The stability ranking of each group, as shown in **A** (Corm growth), **B** (Different tissues), **C** (Drought stress), and **D** (All samples), were presented in line chart. A lower value of the M value indicates more stable expression. An analysis of the pairwise variation (Vn/Vn + 1) between the normalization factors (NFs), denoted as NFn and NFn + 1, were conducted, where n represents the number of genes involved in the NF (**E**). When the pairwise variation value is lower than 0.15 (dot line), the n number would be regarded as optimal number of genes for normalization

The geNorm program was utilized to calculate the M values of 10 candidate RGs, and the stability of each candidate RG was ranked based on its M value. Lower M values indicate more stable expression, designating the corresponding genes as suitable RGs [27]. The samples were categorized into four groups: corm growth stage, different tissues, drought stress, and the overall dataset (all samples). We ranked the candidate RGs based on the average expression stability values (M). The optimal RGs varied depending on experimental conditions. For samples collected at various corm growth stages, *ACY-1* and *CCX4L* were identified as the most reliable RGs (Fig. 4A). *USP25* and *COX10* exhibited the best stability and were considered optimal RGs among different tissues (Fig. 4B). In the case of drought stress treatment, *PIA2* and *Armc8* were deemed the most suitable RGs (Fig. 4C). Lastly, *Armc8* and *COX10* were identified as the most stable RGs for the entire dataset, encompassing all samples (Fig. 4D).

The geNorm algorithm determines the optimal number of RGs by calculating pairwise variation (Vn/n + 1) between consecutive normalization factors, NFn and NFn + 1. According to Vandesompele et al. (2002), when Vn/n + 1 is below the threshold of 0.15, the minimum value of n represents the optimal number of RGs required. In this study, Fig. 4E illustrates that the V2/3 values for corm growth stages and different tissues were both lower than the cutoff value of 0.15. This suggests that using two RGs adequately normalized qRT-PCR gene expression data under these two conditions. However, under drought stress and across all samples, the V3/4 value was lower than 0.15, while the lowest V2/3 value was above 0.15. It suggestes three RGs should be used under these two conditions.

### NormFinder analysis

NormFinder is an algorithm that assesses the expression stability of candidate RGs by considering both intra- and inter-treatment expression variations. Lower expression stability values indicate more stable gene expression [28]. At corm growth stages and in all samples, *PIA2* was identified as the most stable gene, while Armc8 exhibited the highest stability in different tissues and drought stress treatment (Table 4). No wonder, the least stable gene was *β-tubulin* in all the groups.

**Table 4:** The RGs were ranked based on their expression stability values determined using the NormFinder analysis

**Stability Ranking**	**Corm growth**		**Different tissues**		**Drought stress**		**All samples**	
	**Gene**	**Stability value**	**Gene**	**Stability value**	**Gene**	**Stability value**	**Gene**	**Stability value**
1	*PIA2*	0.128	*Armc8*	0.149	*Armc8*	0.264	*PIA2*	0.287
2	*ACY-1*	0.173	*PIA2*	0.222	*COX10*	0.397	*Armc8*	0.318
3	*CCX4L*	0.205	*COX10*	0.361	*CCX4L*	0.401	*CCX4L*	0.429
4	*USP25*	0.365	*CCX4L*	0.47	*PIA2*	0.457	*COX10*	0.488
5	*LYK3*	0.411	*USP25*	0.479	*ACY-1*	0.627	*ACY-1*	0.493
6	*COX10*	0.428	*ACY-1*	0.6	*USP25*	0.673	*USP25*	0.685
7	*Armc8*	0.46	*Actin*	0.772	*LYK3*	1.049	*Actin*	0.933
8	*EF-1α*	0.502	*LYK3*	0.899	*Actin*	1.168	*LYK3*	1.141
9	*Actin*	0.588	*EF-1α*	1.79	*EF1_a*	1.723	*EF-1α*	1.619
10	*β-tubulin*	0.699	*β-tubulin*	2.001	*β-tubulin*	1.782	*β-tubulin*	2.181

### Comprehensive ranking of RGs

**Fig. 5:**
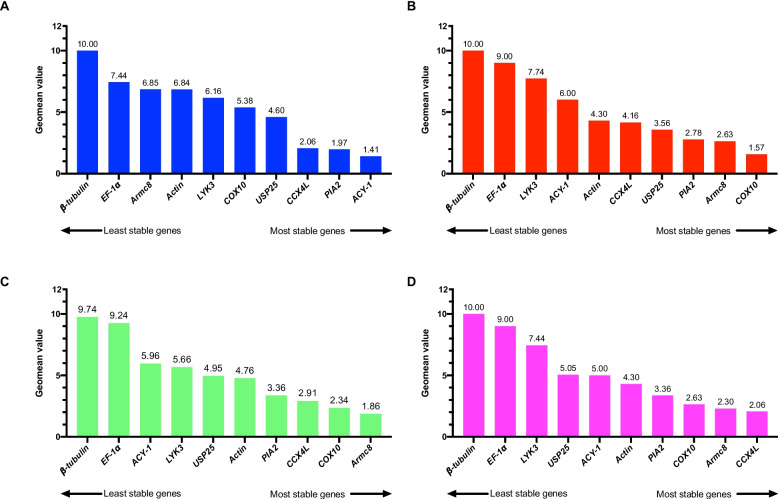
The comprehensive ranking of RGs in four groups using RefFinder program. The four groups are **A** (Corm growth), **B** (Different tissues), **C** (Drought stress), and **D** (All samples). The gene with lower Geomean value is more stable

Upon comparing the outputs of the four methods employed, we noticed some discrepancies in the selection of the most stable RGs. This variance in stability ranking, as observed among Delta Ct, BestKeeper, geNorm, and NormFinder could be attributed to the distinct algorithms utilized by these tools [33, 34]. To obtain a comprehensive analysis of the results, we calculated the geomean of the ranking values by the RefFinder tool [29]. During corm growth, the most stable RGs were *ACY-1*, *PIA2*, and *CCX4L* (Fig. 5A). Among different tissues, the top three most stable RGs were *COX10*, *Armc8*, and *PIA2* (Fig. 5B). Under drought stress, the most stably expressed RGs were *Armc8*, *COX10*, and *CCX4L* (Fig. 5C). Across all samples, *CCX4L*, *Armc8*, and *COX10* were the top three most stable RGs (Fig. 5D). Interestingly, the top three RGs for both drought stress and all samples were the same, although the ranking orders were not necessarily identical. By combining the results from geNorm, the optimal number of RGs for normalization was determined. *ACY-1* and *PIA2* were considered as the optimal multiple RGs for corm growth stages. For different tissues, *COX10* and *Armc8* were identified as the optimal RGs. However, for drought stress and all the samples, three RGs should be selected for normalization. Specifically, *CCX4L*, *COX10*, and *Armc8* were utilized as ideal multiple RGs for normalization in these two situations.

### Validation of the recommended RGs

**Fig. 6:**
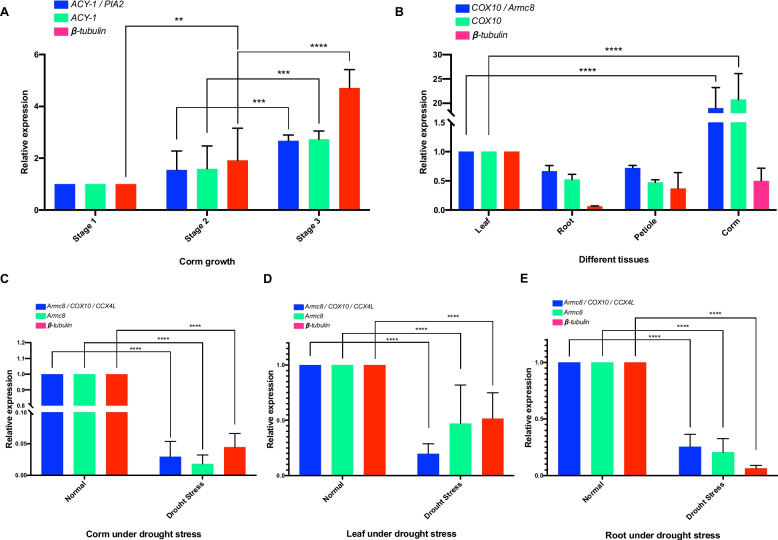
Relative expression of *CeAGPL1* normalized with different sets of RGs. The relative expression of *CeAGPL1* was validated in three corm growth stages (**A**), across four kinds of tissues (**B**), and under drought stress in corm (**C**), leaf (**D**), and root (**E**). Three normalization strategies were used, including multiple top-ranked RGs (Blue color), single top-ranked RG (Green), and the least stable RG (Red). The expression change with significant was indicated in line and asterisk

To validate the selected candidate RGs, we examined the expression profile of the target gene *CeAGPL1*, which is involved in increasing starch content. For each treatment, we employed the optimal multiple RGs chosen from that specific condition, as well as the least stable gene, *β-tubulin*. Additionally, we used the optimal multiple RGs selected from all samples (*ACY-1* and *PIA2* during corm growth; *COX10* and Armc8 within different tissues; and *Armc8*, *COX10*, and *CCX4L* under drought stress) for normalization of the *CeAGPL1* expression. The results revealed interesting patterns in *CeAGPL1* expression. During corm growth, *CeAGPL1* was upregulated continually even based on different RGs (Fig. 6A). However, the expression of the *CeAGPL1* gene was significantly upregulated at stage 2 (60 d) using β-tubulin, whereas the changes were not significant using the other two RGs (*ACY-1* and *PIA2,* or only *ACY-1*). Within different tissues, when normalized with the optimal multiple RGs (*COX10* and *Armc8*) and the most stable gene (*COX10*), *CeAGPL1* expression was significantly increased in corm compared to the leaf (Fig. 6B). However, using the least stable gene (*β-tubulin*) for normalization between different tissues resulted in downregulation of *CeAGPL1*, leading to discrepancies compared to normalization with the optimal multiple RGs. Under drought stress, employing the three normalization methods, *CeAGPL1* expression exhibited a notable decrease across the three types of tissues (Fig. 6C, D and E).

## Discussion

Gene expression analysis plays a crucial role in understanding the growth progress and response mechanisms of plants under various conditions. Several techniques have been developed to investigate gene expression at the transcriptional level, with RT-qPCR being one of the most widely used methods [35]. RT-qPCR allows for accurate and reproducible quantification of transcript abundance of target genes. However, it is essential to perform proper normalization using suitable RGs to ensure a reliable assessment of gene expression in RT-qPCR analysis [36]. There is mounting evidence indicating that the expression levels of commonly used RGs can exhibit substantial variations in specific contexts. Hence, employing specifically selected RGs that are appropriate for the given conditions is a more reliable approach to obtain accurate gene expression measurements in RT-qPCR [37]. With advancements in molecular biology and genomics, taro, a widely grown starchy root crop, has received growing interest in investigating taro at the molecular level [20]. Given the absence of validated RGs reported in taro, it becomes crucial to conduct a thorough validation process to identify a set of stable RGs under specific experimental conditions. In this study, RGs suitable for quantifying the expression level of genes involved in corm growth, different tissues, and drought stress were selected and evaluated (Fig. 1).

Screening the most stable expression gene from transcriptome data have been proved an effective strategy [33, 34, 38]. We adopted 42 transcriptome datasets from taro studies that encompassed corm color, corm growth, and tissue. A total of seven potential novel RGs were identified from the transcriptome data of taro based on specific criteria (Table 1 and Supplementary Table S1). Previous studies have demonstrated the reliability of transcriptomic data in identifying suitable RGs in various species, utilizing similar screening criteria [38, 39]. However, in the case of taro, the stability of *actin*, which were commonly used as an RG for normalizing RT-qPCR data, has not been evaluated under specific experimental conditions [13–15, 20]. Hence, to assess its expression stability under acid stress, *actin* was included as a candidate RG in this study. In addition, two other commonly used RGs, *EF-1α* and *β-tubulin* [40, 41], were employed for validation in these studies. We designed qPCR primers and protocol based on the SYBR dye method with the advantages of ease of use and cost-effectiveness. This choice was made in alignment with our study's principal aim of enhancing accessibility and affordability, ultimately promoting wider participation within taro research community.

The expression levels of these candidate RGs could exhibit significant variations depending on the specific experimental conditions investigated. Among Delta Ct, BestKeeper, geNorm, and NormFinder methods, a slightly divergent pattern in the ranking of expression stability was observed under the same conditions. This finding aligns with previous reports, indicating that variations in results could be attributed to discrepancies in the algorithms and analytical procedures employed by different methods [42–44]. To counteract potential biases and minimize conflicting outcomes resulting from the use of a single method, we used the RefFinder program to perform a comprehensive ranking of the candidate RGs (Fig. 5). The results revealed that the expression stability of these RGs differed under different conditions, emphasizing the necessity of validating RGs specifically within the context of the experimental treatments employed. Notably, *ACY-1*, the gene exhibiting the lowest coefficient of variation (CV) value in the transcriptome data, demonstrated remarkable stability across different stages of corm growth. This finding signifies the consistency between validation using qRT-PCR and transcriptome analysis. It has been previously reported that *ACY-1* plays a role in regulating root growth and stress response in *Nicotiana benthamiana* [45]. Therefore, it is not surprising that *ACY-1* displayed stable expression during corm growth and exhibited relatively lower stability under drought stress conditions in our study. *PIA2* and *CCX4L* were ranked as the second- and third-most stable genes, respectively, during corm growth. Interestingly, *COX10* and *Armc8* were identified as the top three stable genes in the other three groups, including different tissues, drought stress, and all samples. Moreover, the three most stable genes under drought stress conditions (*Armc8*, *COX10*, and *CCX4L*) were consistent with the top three stable genes observed across all the samples. However, there was no single universal RG that consistently ranked among the top three across all experimental groups. The expression stability of RGs varied depending on the specific experimental conditions and treatments being studied. This highlights the importance of selecting appropriate RGs and validating their stability for each experimental scenario to ensure accurate and reliable normalization of gene expression data.

In addition to evaluating the expression stability of candidate RGs and identifying the most stable ones, another crucial aspect of RG validation is determining the optimal number of RGs for proper normalization [46]. Studies have highlighted the necessity of using multiple RGs for accurate assessment of gene expression under specific experimental conditions [47–49]. The rationale behind using multiple RGs lies in the assumption that any experimental errors present would affect all genes expressed in the same sample, assuming they are processed together. Consequently, the individual replicates' experimental errors are averaged across the RGs, and employing the geometric mean of multiple RGs yields a more robust estimate of the overall experimental error compared to using individual RG. The geNorm algorithm is frequently employed to ascertain the optimal number of RGs through analysis of pairwise variation (Vn/n + 1) [27]. For corm growth and different tissue groups, the V2/3 value was below the cut-off of 1.5, indicating that two RGs would suffice to normalize gene expression. Therefore, the recommended RGs for corm growth and different tissues were *ACY-1*/*PIA2* and *COX10*/*Armc8*, respectively (Fig. 5 A and B). However, under drought stress, the V4/5 value was less than 0.15, suggesting the need for at least four RGs to accurately evaluate gene expression. In all the sample groups, the first value that lower than 0.15 was V3/4, indicating that using of three RGs is more accurate (Fig. 4). Given the increased cost and effort associated with using a higher number of RGs, it is reasonable to consider utilizing three RGs (*Armc8*, *COX10*, and *CCX4L*) that demonstrated stability for both drought stress and all samples (Fig. 5C and D).

In our study, three commonly used RGs were employed for stability analysis. However, *EF1_α* and *β-tubulin* were ranked as the least stably expressed genes (Fig. 5). Several recent studies have highlighted that the expression levels of certain traditional RGs can exhibit significant variations under specific experimental conditions, challenging the assumption that they are inherently stable, as previously believed [50, 51]. These findings emphasize the importance of critically re-evaluating the suitability of RGs and highlight the need for careful selection and validation of appropriate RGs tailored to the specific experimental context for accurate and reliable normalization of gene expression data.

To validate the selected RGs, three RG strategies were applied to normalize the expression level of the target gene *CeAGPL1*, which encodes an AGPase large subunit involved in increasing starch content [30, 52]. When normalized using the optimal RGs, except under drought stress, the expression of was notably upregulated in corm growth stage 3, but not in stage 2 (Fig. 6A). In addition, with optimal RGs, the transcriptional level of *CeAGPL1* was significantly higher in corm than in leaves (Fig. 6B). Similar expression trends of *CeAGPL1* were observed in our previous study, where the expression of *CeAGPL1* was accessed by transcriptome [30]. However, when normalized with the least stable RG of *β-tubulin*, overestimation of *CeAGPL1* expression was observed in stage 2, and the expression of *CeAGPL1* in corm was underestimated, although not significantly. The choice of RGs significantly influenced the expression pattern of *CeAGPL1*. This highlights the critical importance of using reliable RGs as a prerequisite for accurate RT-qPCR data analysis of taro under various conditions.

Furthermore, it is important to acknowledge the limitations of the methods used for RNA quantification in this study. Although we primarily employed a spectrophotometer for RNA quantification, it is essential to recognize that this method may have shortcomings in accurately quantifying RNA. Alternative methods, such as a fluorimeter with RNA-specific dyes, would offer greater accuracy in RNA quantification, and future studies may benefit from incorporating these methods to enhance the accuracy of RNA quantification.

## Conclusion

This study highlights the potential of transcriptomic datasets from taro to identify novel candidate RGs for RT-qPCR assays and emphasizes the importance of experimental validation of gene expression stability at diverse stages. Among seven candidate genes with relatively high expression levels and low variance, transcriptomic datasets generated during corm growth and different taro tissues were used for screening. Three commonly used RGs were also included in validation. Through the application of four statistical algorithms (Delta Ct, BestKeeper, geNorm, and NormFinder) and a comprehensive program (RefFinder) for analysis, along with validation using the target gene *ACY-1* and *PIA2* were identified as ideal RGs during corm growth, while *COX10* and Armc8 were suitable for different tissues, *Armc8*, *COX10*, and *CCX4L* were the optimal RGs under drought stress. Significantly, it was observed that *β-tubulin* was not stably expressed in taro. This study represents the first systematic analysis of RGs for RT-qPCR in taro, especially during corm growth. These findings are valuable for future investigations into functional genes related to corm growth in taro and will aid in selecting suitable RGs for starch biosynthesis research in tuberous crop plants during starch accumulation.

### Supplementary Information

Supplementary Material 1.

Supplementary Material 2.

## Data Availability

The datasets analyzed during the current study available from the corresponding author on reasonable request.

## References

[1] Sharma S, Jan R, Kaur R, Riar CS, Nayik GA, Gull A (2020). Taro (Colocasia esculenta). Antioxidants in Vegetables and Nuts - Properties and Health Benefits.

[2] Kreike CM, Van Eck HJ (2004). Lebot V Genetic diversity of taro, Colocasia esculenta (L.) Schott, in Southeast Asia and the Pacific. Theor Appl Genet.

[3] Chaïr H, Traore RE, Duval MF, Rivallan R, Mukherjee A, Aboagye LM, Van Rensburg WJ, Andrianavalona V (2016). Pinheiro de Carvalho MAA, Saborio F et al Genetic Diversification and Dispersal of Taro (Colocasia esculenta (L.) Schott). PLoS One.

[4] Winter KB (2012). Kalo [Hawaiian Taro, Colocasia esculenta (L.) Schott] Varieties: An assessment of nomenclatural synonymy and biodiversity. Ethnobot Res Appl.

[5] Netam U, Thakur P (2022). Kar BSS Morphological characterization of Taro [Colocasia esculenta var. antiquorum (L.) Schott.] Genotypes. Pharm Innov J.

[6] Kawasaki M, Takatsuji A, Taniguchi M, Miyake H (2008). Localization of Casparian Bands and Crystal Cells in Relation to Aluminum Distribution in the Primary Root of Eddo under Aluminum Treatment. Plant Production Science.

[7] Brown AC, Valiere A (2004). The medicinal uses of poi. Nutr Clin Care.

[8] Rosell CM, Matos ME, ArranzE Fernández-BañaresF, RosellCM RodrigoL, PeñaAS, (2015). Market and nutrition issues of gluten-free foodstuff. Advances in the understanding of gluten related pathology and the evolution of gluten-free foods.

[9] Zubair MW, Imran A, Islam F, Afzaal M, Saeed F, Zahra SM, Akhtar MN, Noman M, Ateeq H, Aslam MA (2023). Functional profile and encapsulating properties of Colocasia esculenta (Taro). Food Sci Nutr.

[10] Temesgen M, Retta N (2015). Nutritional potential, health and food security benefits of taro Colocasia esculenta (L.): A Review. Food Science and Quality Management.

[11] Andrade LA, Nunes CA, Pereira J (2015). Relationship between the chemical components of taro rhizome mucilage and its emulsifying property. Food Chem.

[12] Mitharwal S, Kumar A, Chauhan K, Taneja NK. Nutritional, phytochemical composition and potential health benefits of taro (Colocasia esculenta L.) leaves: A review. Food Chem. 2022;383:132406. 10.1016/j.foodchem.2022.132406 35176712

[13] Kim Y-O, Jung S, Kim K, Bae H-J (2013). Role of pCeMT, a putative metallothionein from Colocasia esculenta, in response to metal stress. Plant Physiol Biochem.

[14] Jyothi Lekshmi O, Amrutha P, Jeeva M, Veena S, Sreelatha G, Sujina M, Syriac T (2018). Development of an Efficient Real-time PCR Assay to Accurately Quantify Resistant Gene Analogue Expression in Taro (Colocasia esculenta). Journal of Root Crops.

[15] Wang B, Huang Y, Zhang Z, Xiao Y, Xie J. Ferulic Acid Treatment Maintains the Quality of Fresh-Cut Taro (Colocasia esculenta) During Cold Storage. Front Nutr. 2022;9:884844. 10.3389/fnut.2022.884844 PMC9172584 35685892

[16] Dheda K, Huggett JF, Chang JS, Kim LU, Bustin SA, Johnson MA, Rook GA, Zumla A (2005). The implications of using an inappropriate reference gene for real-time reverse transcription PCR data normalization. Anal Biochem.

[17] Song Y, Hanner RH, Meng B (2021). Genome-wide screening of novel RT-qPCR reference genes for study of GLRaV-3 infection in wine grapes and refinement of an RNA isolation protocol for grape berries. Plant Methods.

[18] He W, Xie R, Li H, Wang Y, Chen Q, Lin Y, Zhang Y, Luo Y, Zhang Y, Tang H (2022). Evaluation of suitable qRT-PCR normalization genes for various citrus rootstocks. Plant Biotechnology Reports.

[19] He F, Dong W, Wei S, Qiu Z, Huang J, Jiang H, Huang S, Liu L (2021). Transcriptome analysis of purple pigment formation in Colocasia esculenta. Biocell.

[20] Dong W, He F, Jiang H, Liu L, Qiu Z. Comparative Transcriptome Sequencing of Taro Corm Development With a Focus on the Starch and Sucrose Metabolism Pathway. Front Genet. 2021;12:771081. 10.3389/fgene.2021.771081 PMC8630585 34858484

[21] Ma R, Xu S, Zhao Y, Xia B, Wang R (2016). Selection and Validation of Appropriate Reference Genes for Quantitative Real-Time PCR Analysis of Gene Expression in Lycoris aurea. Front Plant Sci..

[22] Zhang J, Deng C, Li J, Zhao Y (2020). Transcriptome-based selection and validation of optimal house-keeping genes for skin research in goats (Capra hircus). BMC Genomics.

[23] de Jonge HJM, Fehrmann RSN, de Bont ESJM, Hofstra RMW, Gerbens F, Kamps WA, de Vries EGE, van der Zee AGJ, te Meerman GJ, ter Elst A (2007). Evidence Based Selection of Housekeeping Genes. PLoS ONE.

[24] Subhash N, Mohanan CN, Mallia RJ, Muralidharan V (2004). Quantification of stress adaptation by laser-induced fluorescence spectroscopy of plants exposed to engine exhaust emission and drought. Funct Plant Biol.

[25] Silver N, Best S, Jiang J, Thein SL (2006). Selection of housekeeping genes for gene expression studies in human reticulocytes using real-time PCR. BMC Mol Biol.

[26] Pfaffl MW, Tichopad A, Prgomet C, Neuvians TP (2004). Determination of stable housekeeping genes, differentially regulated target genes and sample integrity: BestKeeper–Excel-based tool using pair-wise correlations. Biotechnol Lett.

[27] Vandesompele J, De Preter K, Pattyn F, Poppe B, Van Roy N, De Paepe A, Speleman F: Accurate normalization of real-time quantitative RT-PCR data by geometric averaging of multiple internal control genes. Genome Biol 2002, 3(7):research0034.0031. 10.1186/gb-2002-3-7-research0034 PMC126239 12184808

[28] Andersen CL, Jensen JL, Ørntoft TF (2004). Normalization of real-time quantitative reverse transcription-PCR data: a model-based variance estimation approach to identify genes suited for normalization, applied to bladder and colon cancer data sets. Cancer Res.

[29] Xie F, Xiao P, Chen D, Xu L, Zhang B: miRDeepFinder: a miRNA analysis tool for deep sequencing of plant small RNAs. Plant Mol Biol 2012. 10.1007/s11103-012-9885-2 22290409

[30] HE Fanglian LL (2022). JIANG Huiping, QIU Zuyang, HUANG Shiyu, DONG Weiqing: Cloning, Bioinformatics and Expression Analysis of ADP-glucose Pyrophosphorylase Gene Family in <i>Colocasia esculenta</i>. Chinese Journal of Tropical Crops.

[31] Livak KJ, Schmittgen TD (2001). Analysis of relative gene expression data using real-time quantitative PCR and the 2(-Delta Delta C(T)) Method. Methods.

[32] Li J, Zhang Z, Xu C, Wang D, Lv M, Xie H (2020). Identification and validation of reference genes for real-time RT-PCR in Aphelenchoides besseyi. Mol Biol Rep.

[33] Pombo MA, Ramos RN, Zheng Y, Fei Z, Martin GB, Rosli HG (2019). Transcriptome-based identification and validation of reference genes for plant-bacteria interaction studies using Nicotiana benthamiana. Sci Rep.

[34] Zhao N, Xu J, Jiao L, Qiu M, Zhang J, Wei X, Fan M. Transcriptome-Based Selection and Validation of Reference Genes for Gene Expression Analysis of Alicyclobacillus acidoterrestris Under Acid Stress. Front Microbiol. 2021;12:731205. 10.3389/fmicb.2021.731205 PMC8430261 34512609

[35] Grätz C, Bui MLU, Thaqi G, Kirchner B, Loewe RP, Pfaffl MW. Obtaining Reliable RT-qPCR Results in Molecular Diagnostics—MIQE Goals and Pitfalls for Transcriptional Biomarker Discovery. Life. 2022;12(3):386. 10.3390/life12030386 PMC8953338 35330136

[36] Bustin S, Nolan T (2017). Talking the talk, but not walking the walk: RT-qPCR as a paradigm for the lack of reproducibility in molecular research. Eur J Clin Invest.

[37] Hruz T, Wyss M, Docquier M, Pfaffl MW, Masanetz S, Borghi L, Verbrugghe P, Kalaydjieva L, Bleuler S, Laule O (2011). RefGenes: identification of reliable and condition specific reference genes for RT-qPCR data normalization. BMC Genomics.

[38] Narsai R, Ivanova A, Ng S, Whelan J (2010). Defining reference genes in Oryza sativausing organ, development, biotic and abiotic transcriptome datasets. BMC Plant Biol.

[39] Zhao J, Yang F, Feng J, Wang Y, Lachenbruch B, Wang J, Wan X (2017). Genome-Wide Constitutively Expressed Gene Analysis and New Reference Gene Selection Based on Transcriptome Data: A Case Study from Poplar/Canker Disease Interaction. Front Plant Sci..

[40] Lin YL, Lai ZX (2010). Reference gene selection for qPCR analysis during somatic embryogenesis in longan tree. Plant Sci.

[41] Kumar K, Muthamilarasan M (2013). Prasad M Reference genes for quantitative real-time PCR analysis in the model plant foxtail millet (Setariaitalica L.) subjected to abiotic stress conditions. Plant Cell Tissue Organ Cult (PCTOC).

[42] Cinar MU, Islam MA, Uddin MJ, Tholen E, Tesfaye D, Looft C, Schellander K (2012). Evaluation of suitable reference genes for gene expression studies in porcine alveolar macrophages in response to LPS and LTA. BMC Res Notes.

[43] de Almeida MR, Ruedell CM, Ricachenevsky FK, Sperotto RA, Pasquali G, Fett-Neto AG (2010). Reference gene selection for quantitative reverse transcription-polymerase chain reaction normalization during in vitro adventitious rooting in Eucalyptus globulus Labill. BMC Mol Biol.

[44] Kałużna M, Kuras A, Puławska J (2017). Validation of reference genes for the normalization of the RT-qPCR gene expression of virulence genes of Erwinia amylovora in apple shoots. Sci Rep.

[45] Chen D, Li J, Jiao F, Wang Q, Li J, Pei Y, Zhao M, Song X, Guo X. ZmACY-1 Antagonistically Regulates Growth and Stress Responses in Nicotiana benthamiana. Front Plant Sci. 2021;12:593001. 10.3389/fpls.2021.593001 PMC8343404 34367193

[46] Chervoneva I, Li Y, Schulz S, Croker S, Wilson C, Waldman SA, Hyslop T (2010). Selection of optimal reference genes for normalization in quantitative RT-PCR. BMC Bioinformatics.

[47] Vandesompele J, Kubista M, Pfaffl MW (2009). Reference gene validation software for improved normalization. Real-time PCR: current technology and applications.

[48] Liang L, He Z, Yu H, Wang E, Zhang X, Zhang B, Zhang C, Liang Z (2020). Selection and validation of reference genes for gene expression studies in Codonopsis pilosula based on transcriptome sequence data. Sci Rep.

[49] Li M, Li X, Wang C, Li Q, Zhu S, Zhang Y, Li X, Yang F, Zhu X. Selection and Validation of Reference Genes For qRT-PCR Analysis of Rhopalosiphum padi (Hemiptera: Aphididae). Front Physiol. 2021;12:663338. 10.3389/fphys.2021.663338 PMC8079785 33935809

[50] Expósito-Rodríguez M, Borges AA, Borges-Pérez A, Pérez JA (2008). Selection of internal control genes for quantitative real-time RT-PCR studies during tomato development process. BMC Plant Biol.

[51] Li L, Li N, Fang H, Qi X, Zhou Y (2020). Selection and Validation of Reference Genes for Normalisation of Gene Expression in Glehnia littoralis. Sci Rep.

[52] Petreikov M, Shen S, Yeselson Y, Levin I, Bar M, Schaffer AA (2006). Temporally extended gene expression of the ADP-Glc pyrophosphorylase large subunit (AgpL1) leads to increased enzyme activity in developing tomato fruit. Planta.

