# Supplementary material for: Transcriptome-based identification and validation of reference genes for corm growth stages, different tissues, and drought stress in Taro (Colocasia esculenta)
Source: BMC Plant Biol. 2024 May 30;24:478. doi: 10.1186/s12870-024-05199-x (PMC11137888; doi:10.1186/s12870-024-05199-x)
Supplement: Supplementary file 2 — Supplementary Material 2. [file 12870_2024_5199_MOESM2_ESM.pdf]

Supplementay Figure

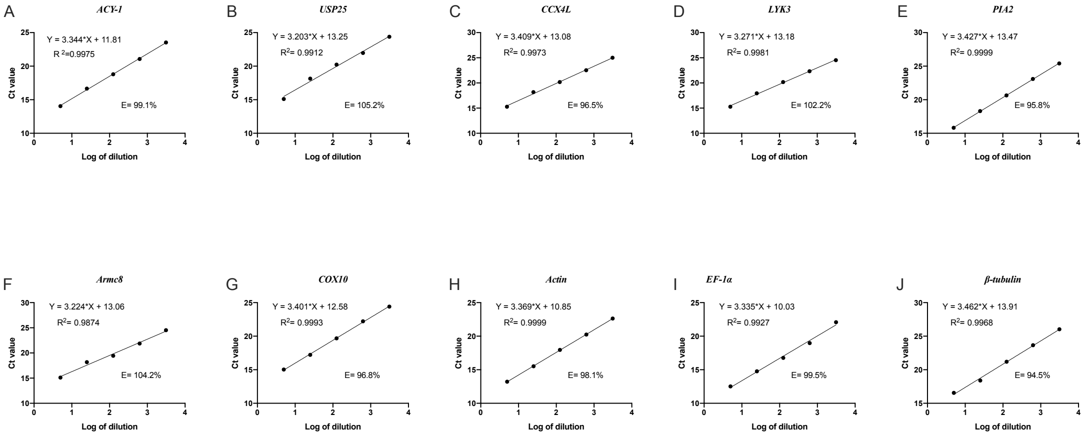

Supplementary Figure 1 Amplification efficiency analysis of 10 candidate RGs

Standard curves of the correlation between the Ct values and template concentrations were depicted. The amplification efficiency (E) was calculated based on the slope of the standard curve. The ten candidate RGs were *ACY-1* (A), *USP25* (B), *CCX4L* (C), *LYK3* (D), *PIA2* (E), *Armc8* (F), *COX10* (G), *Actin* (H), *EF-1α* (I), and *β-tubulin* (J).
